# Application of machine learning algorithms in predicting carotid artery plaques using routine health assessments

**DOI:** 10.3389/fcvm.2024.1454642

**Published:** 2024-09-23

**Authors:** Yuting Wei, Junlong Tao, Yifan Geng, Yi Ning, Weixia Li, Bo Bi

**Affiliations:** ^1^ School of Public Health, Hainan Medical University, Haikou, Hainan, China; ^2^ Key Laboratory of Tropical Translational Medicine of Ministry of Education, Hainan Medical University and Hainan Academy of Medical Sciences, Haikou, Hainan, China; ^3^ The First Affiliated Hospital, Hainan Medical University, Haikou, Hainan, China; ^4^ The Key Lab of Tropical Cardiovascular Diseases Research of Hainan Province, Haikou, Hainan, China

**Keywords:** carotid plaque, cardiovascular diseases, risk prediction, machine learning, healthcare

## Abstract

**Background:**

Cardiovascular diseases (CVD) constitute a grave global health challenge, engendering significant socio-economic repercussions. Carotid artery plaques (CAP) are critical determinants of CVD risk, and proactive screening can substantially mitigate the frequency of cardiovascular incidents. However, the unequal distribution of medical resources precludes many patients from accessing carotid ultrasound diagnostics. Machine learning (ML) offers an effective screening alternative, delivering accurate predictions without the need for advanced diagnostic equipment. This study aimed to construct ML models that utilize routine health assessments and blood biomarkers to forecast the onset of CAP.

**Methods:**

In this study, seven ML models, including LightGBM, LR, multi-layer perceptron (MLP), NBM, RF, SVM, and XGBoost, were used to construct the prediction model, and their performance in predicting the risk of CAP was compared. Data on health checkups and biochemical indicators were collected from 19,751 participants at the Beijing MJ Health Screening Center for model training and validation. Of these, 6,381 were diagnosed with CAP using carotid ultrasonography. In this study, 21 indicators were selected. The performance of the models was evaluated using the accuracy, sensitivity, specificity, positive predictive value (PPV), negative predictive value (NPV), F1 score, and area under the curve (AUC) value.

**Results:**

Among the seven ML models, the light gradient boosting machine (LightGBM) had the highest AUC value (85.4%). Moreover, age, systolic blood pressure (SBP), gender, low-density lipoprotein cholesterol (LDL-C), and total cholesterol (CHOL) were the top five predictors of carotid plaque formation.

**Conclusions:**

This study demonstrated the feasibility of predicting carotid plaque risk using ML algorithms. ML offers effective tools for improving public health monitoring and risk assessment, with the potential to improve primary care and community health by identifying high-risk individuals and enabling proactive healthcare measures and resource optimization.

## 1 Introduction

The “China Cardiovascular Health and Disease Report 2021” stated that with the developing society and economy, changing national lifestyle, the aging of the population and the acceleration of its urbanization process, and unhealthy lifestyles among residents have become increasingly prominent; thus, the prevalence and incidence continue to rise in 2019. Rural and urban CVD deaths accounted for 46.74% and 44.26% of the total deaths, respectively, and CVD accounted for two out of every five deaths. It is estimated that the number of CVD patients in China is 330 million, and in 2019, the total hospitalization cost of cardiovascular and cerebrovascular diseases in China was 313.366 billion yuan, which poses a major burden on the economy and medical resources (1).

Assessing the epidemiological burden of carotid atherosclerosis can serve as a basis for preventing and managing CVD. Song et al. (2) conducted a systematic review, meta-analysis, and modeling study. Carotid plaque, a characteristic manifestation of atherosclerosis, is found in quantifying the prevalence of atherosclerosis. The proportion of carotid plaque is estimated to be 21.1% (13.2–31.5), equivalent to 815.76 million patients, representing a 58.97% increase since 2000. The Western Pacific region has the largest proportion [240.7 million of 72,525 million patients (33.20%)]. Carotid plaque is a major global health concern, with substantial implications for the prevalence of cardiovascular diseases. Effective prevention and management strategies, including early screening, are essential to mitigate its impact on individuals, families, and societal health. Early detection can significantly reduce the risk associated with cardiovascular conditions and alleviate the broader social and economic burden. Despite its importance, there is currently no national epidemiological survey targeting carotid plaque in China. This oversight might stem from the fact that carotid ultrasound, the primary screening tool, is often viewed more as a preventive measure than a definitive diagnostic tool for severe illnesses (3, 4). Given these circumstances, there is a critical need to develop cost-effective screening methods to facilitate the early identification and management of carotid plaque.

With the advancement of medical technology and computer science, ML has been widely used in the field of medicine. ML can effectively handle large-scale, high-dimensional data, and automatically extract complex patterns and correlations, surpassing traditional statistical methods. Through iterative optimization of massive data, ML models have strong generalization capabilities, enabling them to adapt to complex real situations. In the analysis of carotid plaques, researchers have developed various predictive models using ML, aiming to predict plaque characteristics and differentiate plaque components. Cilla et al. (5) used CT angiography image group features combined with ML models to effectively distinguish vulnerable and non-vulnerable carotid plaques. Zhang et al. (6) developed a high-risk plaque prediction model based on MRI, using radiomic features to differentiate symptomatic and asymptomatic plaques, with more accuracy than traditional methods. However, although these imaging-based models have high predictive efficiency, they still face resource-intensive challenges in widespread screening.

Comparatively, using regular physical examinations and biochemical indicators as features of the ML model, is a more practical method to conveniently collect data without generating additional costs. This study aims to develop an ML model based on existing indicators to predict carotid artery plaques in an economically efficient and accurate manner, thereby improving the efficiency of clinical cardiovascular risk assessment. We hope that this model can effectively enhance the efficiency of cardiovascular risk assessment in a clinical setting, providing new ideas for early screening and prevention. At the same time, this endeavor will lay the foundation for further exploration of the potential of ML in the prevention of cardiovascular diseases and promote the development of personalized medicine.

## 2 Materials and methods

### 2.1 Study design and data resource

The data used for the ML model in this study were obtained from the physical examination data of Beijing MJ Healthcare from January 2017 to December 2022. The inclusion criteria were as follows: (1) age ≥18 years, and (2) patients without coronary heart disease, stroke, heart disease, cancer, or other serious diseases. It is worth mentioning that, 5,019 participants had missing values for over 30% of potential predictors, and to ensure the authenticity of the data, these participants were excluded from the analysis. Physical examination data were collected from 19,751 patients, including 6,381 patients with carotid plaques and 13,370 normal patients. The dataset was then split into a training set (13,826 participants) and a test set (5,925 participants) at a 7:3 ratio. Subsequently, data normalization was performed, followed by developing, training, and evaluating a machine learning model to ensure the reliability and accuracy of predictions for carotid plaques.

**Figure 1:**
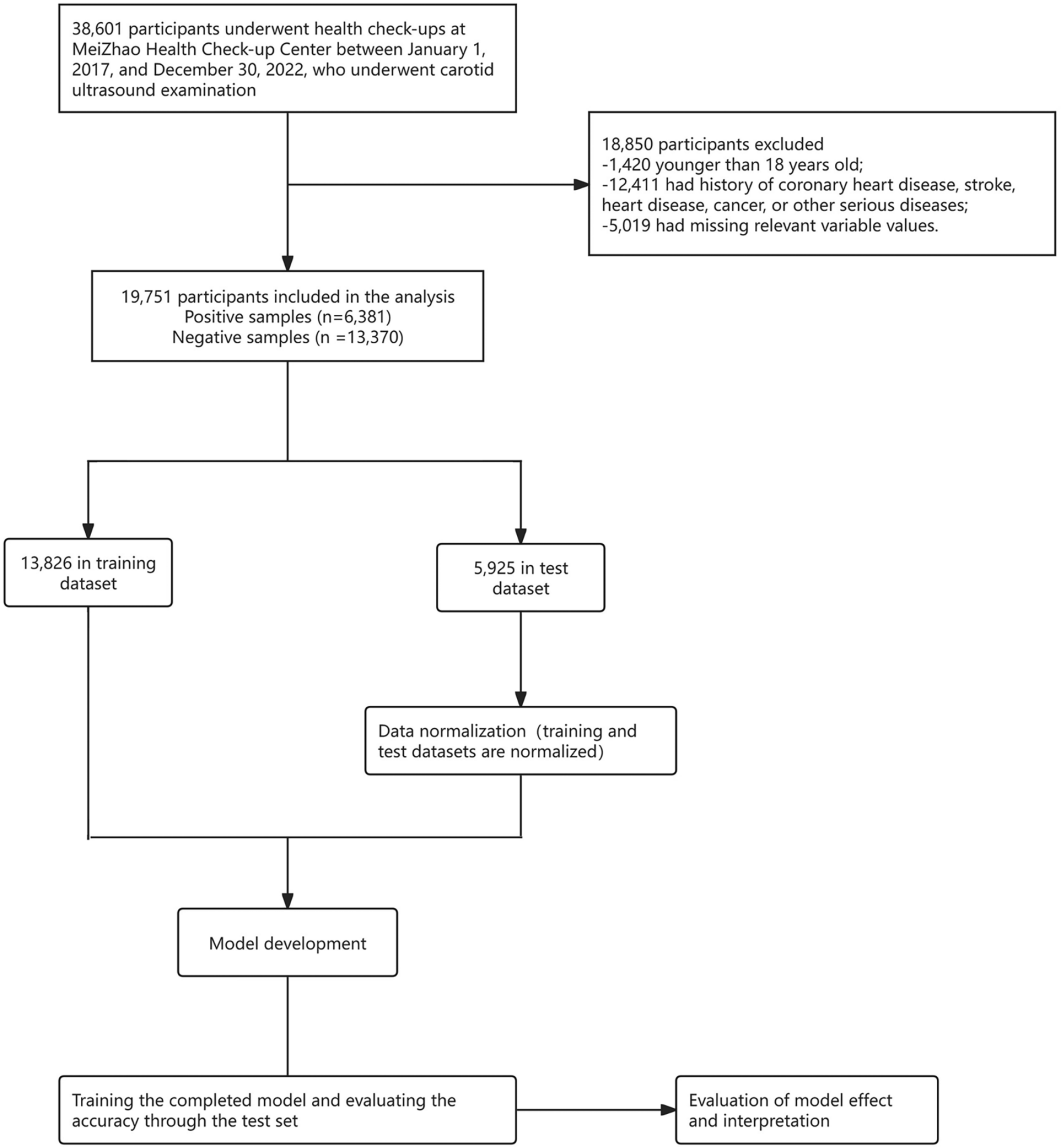
The flow diagram of data processing and model building process.

The data processing and model building processes of ML are displayed in Figure 1.

### 2.2 Quality control and ethics

In our study, we selected a reputable physical examination institution with standardized protocols for ultrasound examinations, ensuring data consistency and reliability. We rigorously screened the data to remove any incomplete records and confirmed the institution's operational and equipment calibration details, enhancing our study's quality. The diagnosis of carotid plaque was based on an ultrasound examination. Strict confidentiality was maintained for all the personal information. Ethical approval was granted by the Institutional Review Board of Hainan Medical University (approval ID: HYLL-2023-449), and all participant data were anonymized. Informed consent was obtained from each participant before administering the questionnaire and providing healthcare services.

### 2.3 Feature selection

All the potential factors associated with carotid plaque reported in recent studies were considered. Considering the availability of variables in the database, 21 variables were extracted according to studies by Fan et al. (7) and Wu et al. (8), including (1) demographic characteristics: gender and age; (2) physical examination indicators: waistline (WaWidth), hip circumference (HipWidth), body mass index (BMI), systolic blood pressure (SBP), and diastolic blood pressure (DBP); (3) laboratory examination indicators: total cholesterol (CHOL), triglycerides (TG), high-density lipoprotein cholesterol (HDL-C), low-density lipoprotein cholesterol (LDL-C), fasting blood glucose (FBG), alanine transaminase (ALT), aspartate aminotransferase (AST), direct bilirubin (DBil), total bilirubin (TBil), alkaline phosphatase (ALP), uric acid (UA), and gamma-glutamyl transferase (GGT); (4) questionnaire survey: alcohol consumption and smoking status. All variables are presented in Table 1.

**Table 1:** Characteristics of study population.

Characteristic	CaroNormal		*p*-value^b^
	Negative, *N* = 13,370^a^	Positive, *N* = 6,381^a^	
Age (Years)	43 ± 9	57 ± 11	<0.001
Sex			<0.001
Male	6,711 (50%)	4,140 (65%)	
Female	6,659 (50%)	2,241 (35%)	
Smoke			<0.001
No	10,870 (81%)	4,384 (69%)	
Yes	2,500 (19%)	1,997 (31%)	
Drink			<0.001
No	10,835 (81%)	4,666 (73%)	
Yes	2,535 (19%)	1,715 (27%)	
BMI (kg/m2)	23.8 ± 3.2	24.7 ± 3.0	<0.001
WaWidth (cm)	80 ± 10	84 ± 9	<0.001
HipWidth (cm)	95.9 ± 5.9	96.5 ± 5.8	<0.001
CHOL (mmol/L)	4.60 ± 0.78	4.74 ± 0.87	<0.001
HDL-C (mmol/L)	1.43 ± 0.34	1.36 ± 0.33	<0.001
LDL-C (mmol/L)	3.05 ± 0.73	3.20 ± 0.82	<0.001
TG (mmol/L)	1.16 ± 0.54	1.34 ± 0.55	<0.001
FBG (mmol/L)	5.39 ± 0.45	5.63 ± 0.51	<0.001
UA (umol/L)	315 ± 81	334 ± 78	<0.001
TBil (umol/L)	11.6 ± 4.2	12.1 ± 4.1	<0.001
DBil (umol/L)	4.24 ± 1.29	4.39 ± 1.28	<0.001
ALP (U/L)	62 ± 16	67 ± 16	<0.001
AST (U/L)	17.3 ± 4.1	18.6 ± 4.2	<0.001
ALT (U/L)	18 ± 8	19 ± 8	<0.001
GGT (U/L)	19 ± 10	21 ± 10	<0.001
SBP (mmHg)	114 ± 14	122 ± 15	<0.001
DBP (mmHg)	70 ± 10	74 ± 10	<0.001

^a^ Mean ± SD; *n* (%).

^b^ Welch two sample *t*-test; Pearson's Chi-squared test.

The outcome was defined as the presence or absence of carotid plaque on the carotid artery ultrasound examination. Specifically, the common carotid arteries, bifurcation, and external and internal carotid arteries were examined on each side by experienced sonographers operating a Doppler ultrasound system (Sonoscape S50, China) with a linear 7.5 MHz probe under standardized protocols. The distance between the lumen-intima echo's leading edge and the media-adventitia echo's leading edge was defined as the carotid intima-media thickness. Based on the Chinese Health Checkup Guidelines for Carotid Artery Ultrasonography (9), patients are considered positive for carotid artery plaques if the perpendicular distance from the leading edge of the intima-lumen interface to the leading edge of the media-adventitia interface exceeds 1.5 mm. This distance should be at least 0.5 mm greater than the surrounding normal values or should exceed the surrounding normal values by more than 50%. Additionally, patients exhibiting localized structural changes protruding into the lumen were also classified as positive for CAP.

### 2.4 ML algorithms

Seven predictive models were used to develop the risk models based on the extracted features to predict the risk of CAP formation.

Logistic regression (LR): LR analyzes datasets with one or more independent variables to predict a dichotomous outcome using a logistic function. This S-shaped function maps any real number into a probability between 0 and 1. LR has low computational costs and performs well with limited data (10).

Support vector machine (SVM): SVM is used for classification and regression. It finds a hyperplane that best separates classes by maximizing the margin between them. The closest points to the hyperplane, called support vectors, are crucial for model construction. SVM enhances the generalization ability for new data (11).

Random forest (RF): RF is an ensemble method that creates multiple decision trees from bootstrapped datasets and random subsets of variables. Trees vote for the final prediction. RF is flexible, easy to use, and performs well without extensive tuning (12).

Light gradient boosting machine (LightGBM): LightGBM is an efficient gradient boosting framework for large-scale data, known for its fast training, low memory usage, and ability to handle categorical features. It supports parallel and GPU learning, built-in cross-validation, and custom objective functions (13).

Extreme gradient boosting (XGBoost): XGBoost is a gradient boosting implementation known for efficiency, performance, and scalability. It excels in handling large datasets, reducing overfitting through regularization, and simplifying model tuning with built-in cross-validation and feature importance assessment. It prunes trees optimally and handles missing values effectively (14).

Naive Bayes model (NBM): NBM is a probabilistic classifier based on Bayes’ theorem with strong independence assumptions between features. It is suitable for high-dimensional datasets and popular in text categorization. NBM calculates conditional probability to make predictions (15).

The multi-layer perceptron (MLP) neural network: MLP is a feedforward Artificial Neural Network (ANN) with at least three layers (input, hidden, output). Neurons use non-linear activation functions, and the network is trained using backpropagation. MLP can handle non-linearly separable data, unlike single-layer perceptrons (16).

### 2.5 Model performance assessment

In this study, we randomly divided the enrolled patients into two groups: a training set (70%) and a validation set (30%). The training set is mainly used for model training, while the validation set is used to fine-tune model parameters and select the best performing model. After this division, we organized and preprocessed the collected data. Data normalization was performed, and features with very low variance, low correlation with the target label, or high interrelations were removed to enhance the quality of the dataset. We adopted a 10-fold cross-validation strategy for the training set, repeated ten times to optimize of hyperparameters for seven classification algorithms. This optimization was achieved using grid search techniques to find the best parameter combinations. After training and modeling data with these algorithms, we evaluated the predictions of each model. The final model score was determined by calculating the average accuracy over multiple iterations. Furthermore, the model with the best AUC was selected as the baseline for this study, resulting in the final predictive model. All ML algorithms were operated in Python 3.10.

### 2.6 Validation

The receiver operating characteristic (ROC) curve is a visual tool for evaluating classification methods/models (17). Its basic principle involves sorting the predicted values for cases and non-cases from the smallest to the largest, thus creating a series of cut-off values. For each cut-off value, the corresponding sensitivity and specificity can be calculated. The point closest to the top-left corner of the coordinate axis can simultaneously satisfy the relatively optimal sensitivity and specificity of the screening test, which is considered the best cut-off value. The area under the curve (AUC) is the area under the ROC curve. The ROC curve is a curve, whereas the AUC is a numerical value used to quantify the performance of an indicator/model, which can be used for performance comparison between two indicators/models. The calibration curve is a tool used to assess the consistency between the probabilities output by predictive models and the actual outcomes, by comparing the predicted probabilities with the actual results. The *x*-axis (horizontal axis) represents the probability values predicted by the model, while the *y*-axis (vertical axis) represents the frequency or proportion of the observed actual outcomes. Ideally, a perfectly calibrated model's calibration curve would follow the diagonal line (i.e., y = x), indicating that the model's predicted probabilities are entirely consistent with the actual occurrence probabilities. Therefore, its shape and position can reveal the model's predictive bias and consistency (18). Decision Curve Analysis (DCA) is a commonly used method for evaluating and comparing the performance of predictive models (19). It integrates both the predictive accuracy and practical utility of the models, aiding in the assessment of the economic benefits and risk-reward trade-offs of different models within specific threshold ranges.

Accuracy is typically defined as the ratio of correct predictions to the total number of predictions. It is a standard metric for evaluating the performance of classification models, particularly when the target variable is binary or multi-classification. Accuracy can be expressed using the following formula:

Accuracy=TP+TN/(TP+FP+TN+FN)×100%

where TP, TN, FP, and FN denote true positive, true negative, false positive, and false negative, respectively.

Sensitivity, also known as the true positive rate or recall, is a statistical measure that quantifies the ability of a model to identify positive cases among all actual positive cases correctly. It is essential in medical testing and other binary classification tasks, where the cost of missing a positive case (such as a disease) can be high.

Sensitivity=TP/(TP+FN)×100%

In the context of model specificity, specificity refers to the ability of a model to identify negative examples (non-target classes) correctly. It measures the proportion of all actual negative cases that the model correctly predicts to be negative. Specificity is critical in cases where the cost of incorrectly predicting a negative case as a positive case (a false positive) is high.

Specificity=TN/(TN+FP)×100%

The positive predictive value (PPV), also known as precision, is a measure of the accuracy of a diagnostic test or performance of a classification model. It represents the proportion of positive test results that are truly positive. In other words, it measures the likelihood that, the result is actually positive when the model predicts a positive result.

PPV=TP/(TP+FP)×100%

The negative predictive value (NPV) is a measure used to assess the performance of a diagnostic test or classification model. It represents the proportion of negative test results that are truly negative. Essentially, NPV indicates the likelihood that when the model predicts a negative result, that result is actually negative.


NPV=TN/(TN+FN)×100%

The F1 score is a measure of a test's accuracy that considers both the precision (or PPV) and the recall (or sensitivity) of the test. It is the harmonic mean of precision and recall, balancing the two when they are uneven.

F1score=2×(PPV×Sensitivity)/(PPV+Sensitivity)

### 2.7 Model interpretation

Shapley Additive exPlanations (SHAP) represent an advanced interpretive method derived from cooperative game theory, which utilizes Shapley values to distribute an ML model's output among its input features (20). This technique ensures a fair contribution assessment by averaging the incremental impact of each feature across all potential combinations, offering a reliable and precise elucidation of the model's predictions. By incorporating SHAP into predictive modeling, it is possible to gain an insightful comprehension of feature impacts, thus bolstering the transparency and intelligibility of intricate models, particularly in critical decision-making contexts. Unlike the conventional interpretive approaches, which merely gauge feature significance, SHAP provides a nuanced perspective on the relationship between features and their predictive outcomes, thereby addressing the limitations of traditional methods. SHAP values, computed for each feature per individual prediction, quantify the extent to which each feature sways the prediction positively or negatively toward the final result.

## 3 Results

### 3.1 Baseline characteristics

**Figure 2:**
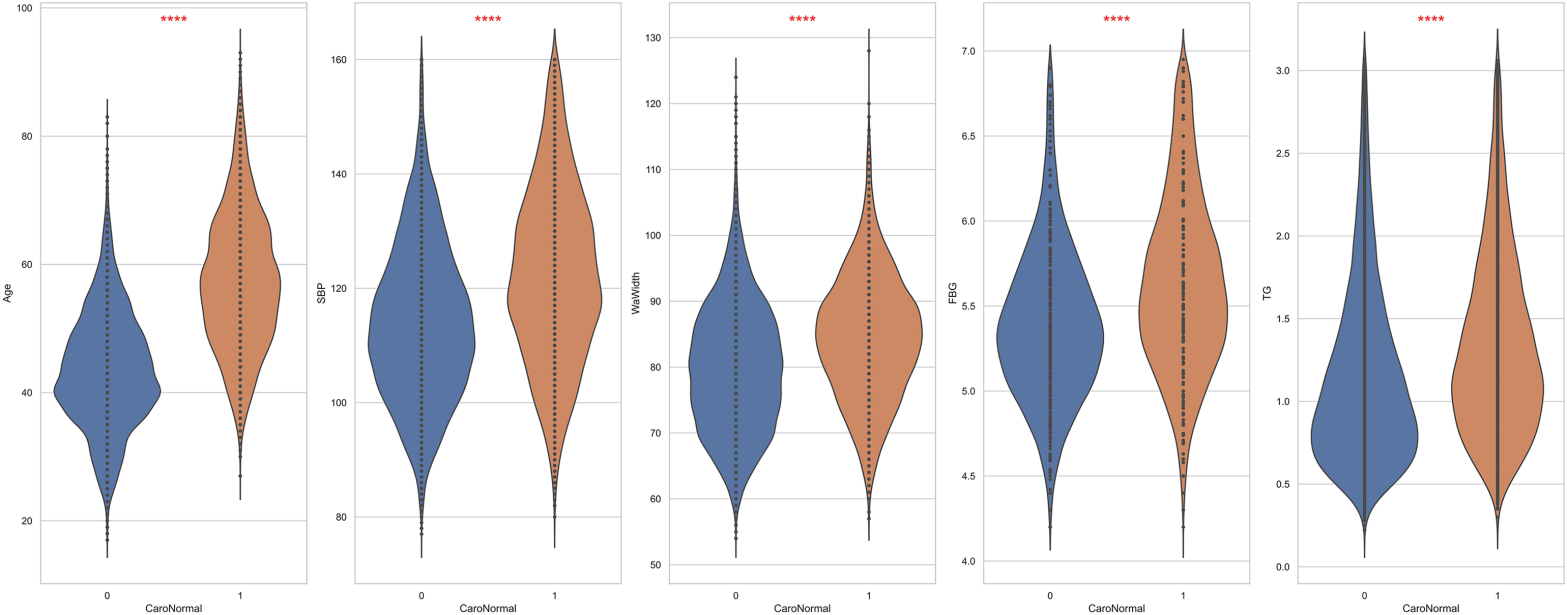
The top 5 features exhibit the most significant differences between patients and non-patients with carotid plaque. ****: *p* < 0.0001.

This study included 19,751 participants: 10,851 males and 8,900 females. Among the participants, 6,381 were diagnosed with carotid plaques, with an average age of 57 ± 11 years, whereas those without carotid plaques had an average age of 43 ± 9 years. Additionally, compared to the control group, the case group had higher average BMI (24.7 ± 3.0 vs. 23.8 ± 3.2, *p* < 0.001), waistline (84 ± 9 vs. 80 ± 10, *p* < 0.001), hip circumference (96.5 ± 5.8 vs. 95.9 ± 5.9, *p* < 0.001), CHOL levels (4.74 ± 0.87 vs. 4.60 ± 0.78, *p* < 0.001), and HDL-C levels (1.36 ± 0.33 vs. 1.43 ± 0.34, *p* < 0.001). All included variables were presented as mean (SD), and a two-sample *t*-test showed significant differences between the two groups (Table 1). Figure 2 demonstrates the violin plots of the five features with the most significant differences. Overall, this chart clearly shows the distribution differences in age, SBP, WaWidth, FBG, and TG between the two groups. Patients with CAP generally exhibit higher values in these variables, suggesting a high correlation between these variables and the formation of CAP.

### 3.2 Comparison of model performance

Table 2 presents a performance comparison of seven ML models on the test set: LightGBM, LR, MLP, NBM, RF, SVM, and XGBoost. Evaluation metrics included accuracy, sensitivity, specificity, PPV, NPV, F1 score, and AUC, comparison of the seven constructed ML models revealed that the LightGBM model outperformed the other algorithms, with an AUC value of 0.8541. LR followed closely in terms of predictive accuracy with an AUC of 0.8460. In contrast, the NBM showed the poorest performance (AUC 0.7966). To statistically validate the observed performance differentials between models, the Delong test was employed—a methodology that rigorously compares the AUC values of two ROC curves by assessing the null hypothesis that both AUCs are equal. The derivation of *p*-values less than 0.05 across all pairwise comparisons of the models unambiguously indicates that the performance disparities are statistically significant. This outcome not only validates the relative ranking of model efficacy, as identified through AUC, but also reinforces the importance of model selection based on empirical evidence in the pursuit of optimal predictive performance.

**Table 2:** Comparison of performance of seven machine learning methods.

Model	Accuracy	Sensitivity	Specificity	PPV	NPV	F1 score	AUC
LightGBM	0.7946	0.5952	0.8920	0.7290	0.8168	0.6553	0.8541
LR	0.7967	0.5926	0.8963	0.7361	0.8184	0.6566	0.8460
MLP	0.7779	0.5689	0.8800	0.6982	0.8070	0.6270	0.8310
Naive bayes	0.7366	0.6772	0.7705	0.5866	0.8258	0.6243	0.7966
RF	0.7953	0.5874	0.8968	0.7354	0.8166	0.6531	0.8428
SVM	0.7933	0.5499	0.9121	0.7533	0.8059	0.6357	0.8291
XGBoost	0.7833	0.5849	0.8802	0.7045	0.8128	0.6491	0.8341

**Figure 3:**
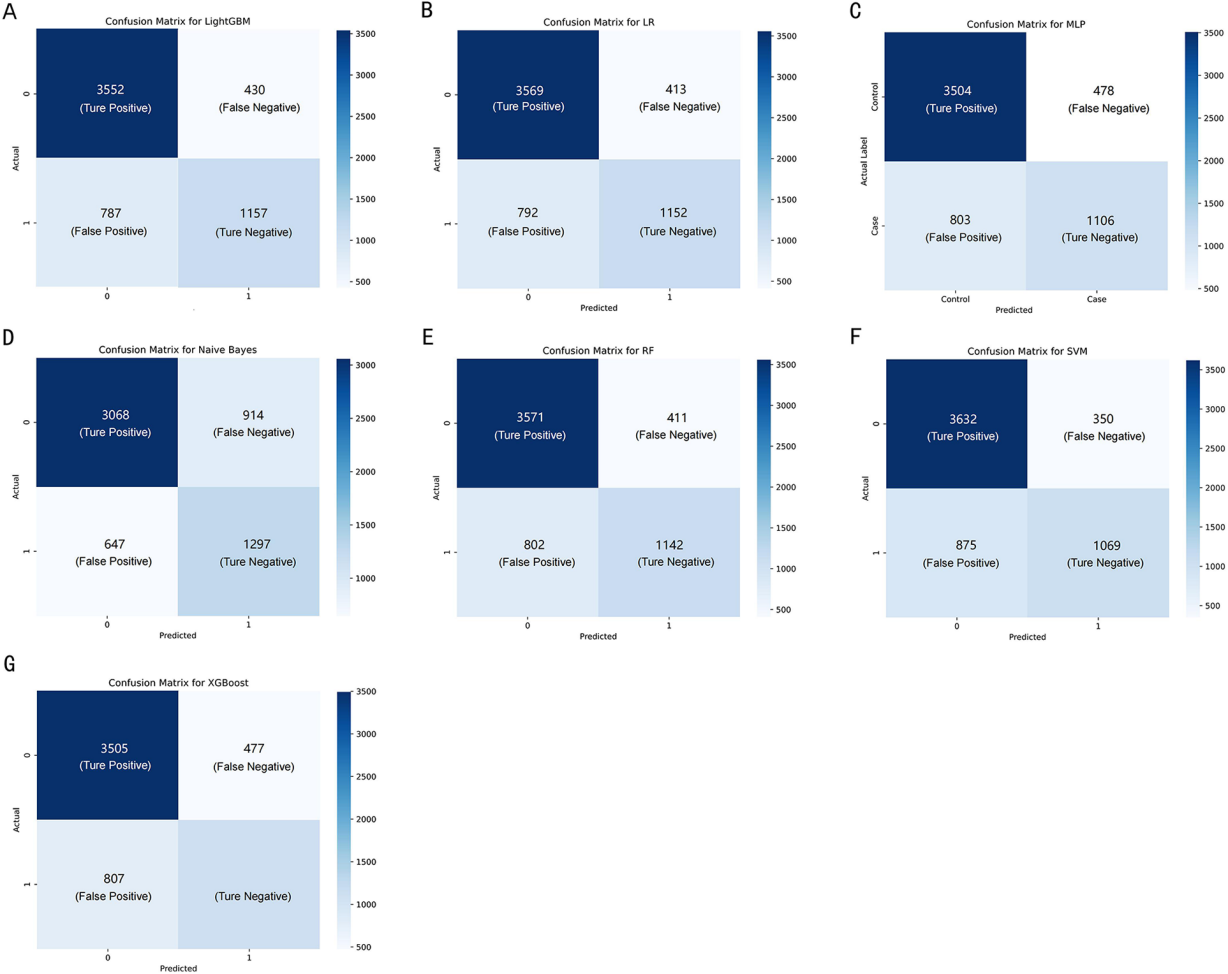
The confusion matrix of the seven machine learning models, including **(A–G)** LightGBM, LR, MLP, NBM, RF, SVM, and XGBoost, respectively.

Confucian matrices were generated to evaluate the performance of the classification models (Figure 3). Each matrix was a 2 × 2 table, with the rows representing the actual classes and the columns representing the classes predicted by the model. The cell in the top-left corner represented the number of TP, indicating correct predictions of the positive class. The bottom-right cell denoted the number of TN, suggesting the correct predictions of the negative class. Conversely, the top-right cell specified FP, and the bottom-left cell indicated FN, representing types of incorrect predictions. These matrices provided a foundation for calculating further performance metrics, such as accuracy, precision, and recall.

**Figure 4:**
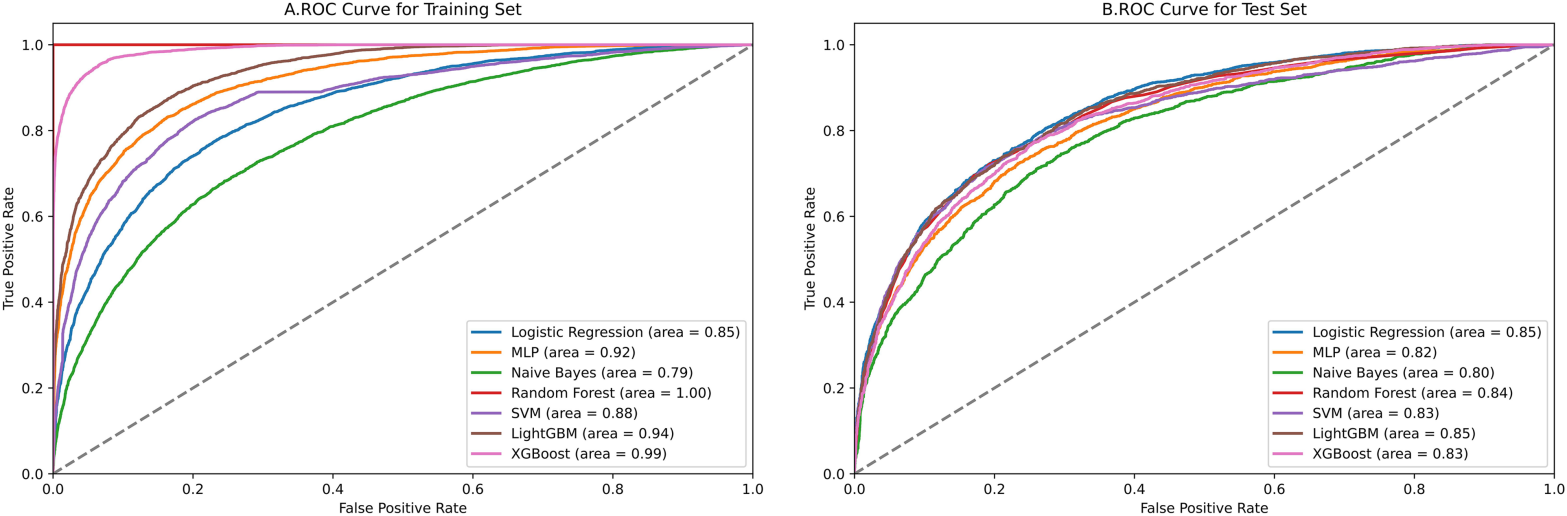
ROC curves of all models.

**Figure 5:**
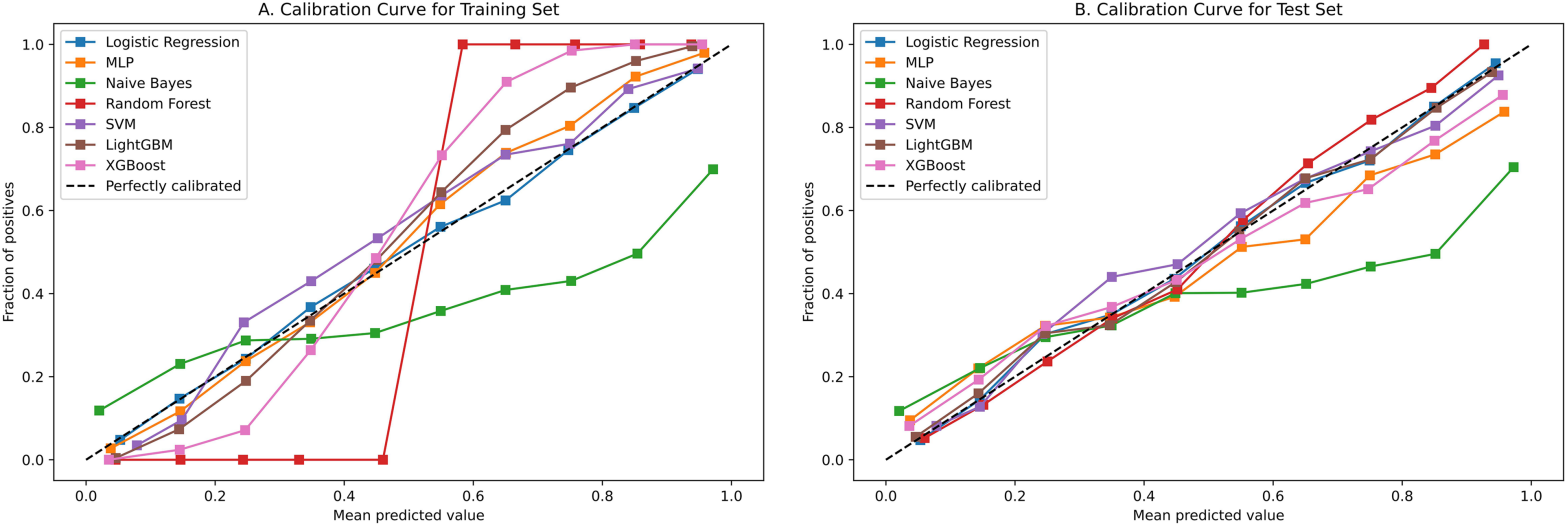
Calibration curve of all model.

**Figure 6:**
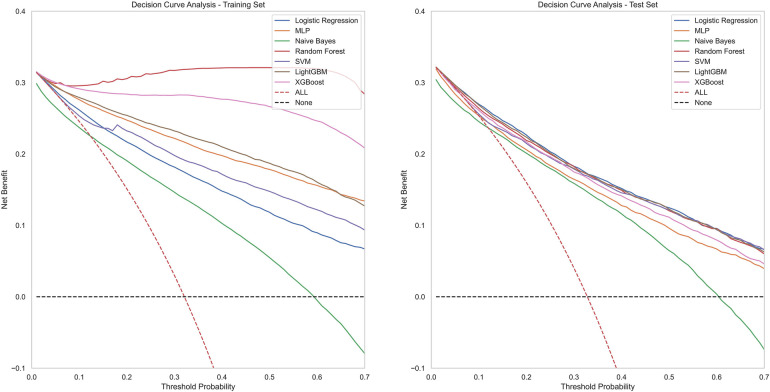
DCA curves of all model.

Figure 4 contains ROC curves for various ML models on training and test sets, illustrating their performance in classifying TP and TN cases. The AUC values, ranging from 0.80 to 0.85, indicated a high level of performance across all models. Exclusively, the LightGBM and LR models exhibited the highest AUC of 0.85, suggesting that they had the best performance in terms of sensitivity and specificity. The NBM had the lowest AUC at 0.80. Figure 5 presents calibration curves for two datasets, providing a visual assessment of the calibration of different models. It can be observed that the LightGBM model's curves for both the training and test sets are close to the dashed line, indicating that the predicted values of this model closely match the actual outcomes, signifying good calibration. Figure 6 displays DCA curves for two datasets, showing that the LightGBM algorithm performs superiorly across different threshold levels. According to these results, the LightGBM demonstrated the best overall performance.

**Figure 7:**
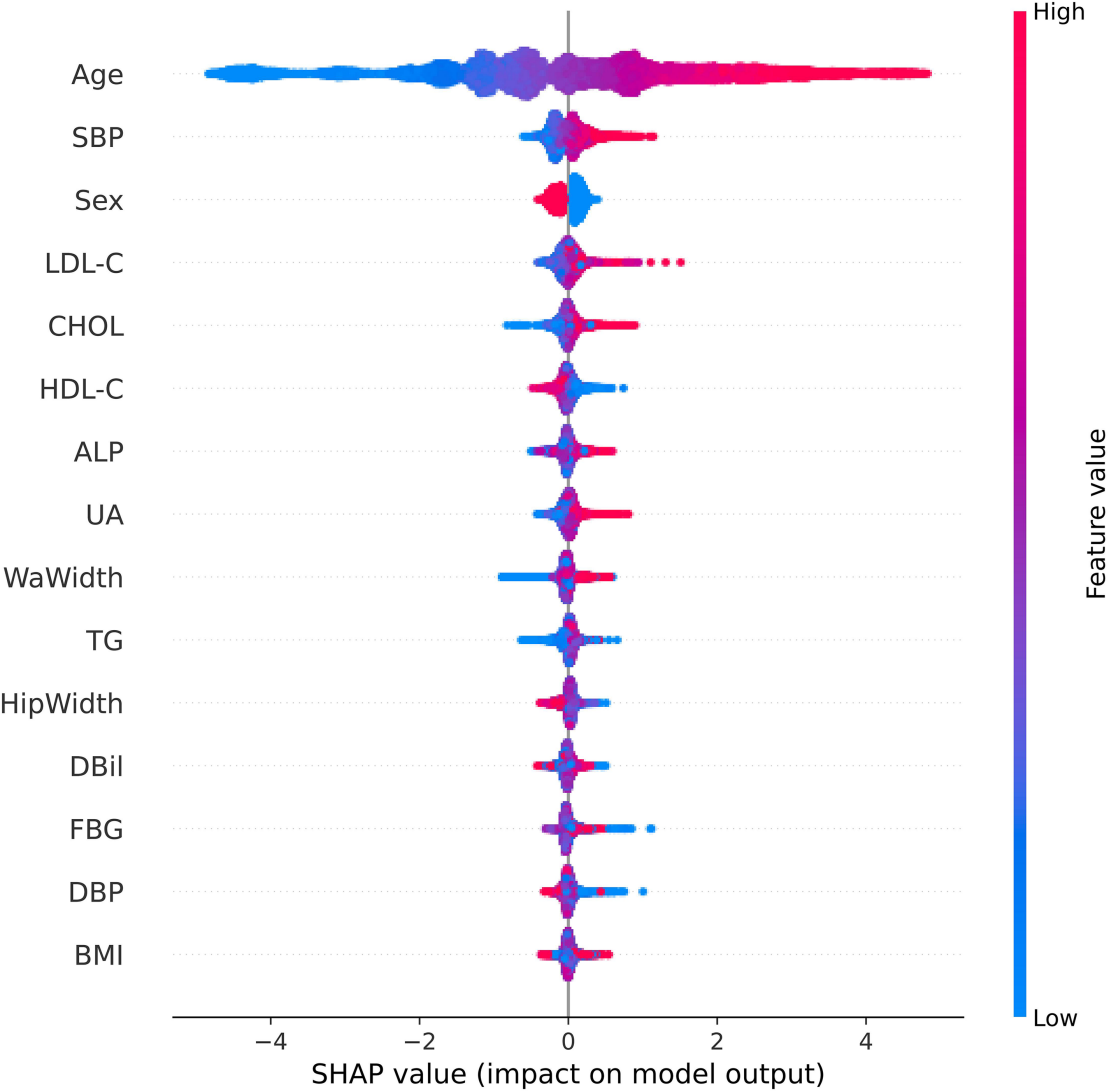
SHAP summary chart of the important risk factors. Each dot represents a sample, with red denoting a high feature value and blue indicating a low value. A higher SHAP value specifies a higher risk of incident carotid plaque.

Based on the LightGBM model's performance, we calculated each feature's importance according to the absolute SHAP values, with the negative and positive contributions of the feature represented in blue and red, respectively. The position of the dot on the horizontal axis indicated the SHAP value associated with that feature for that particular data point. A higher SHAP value on the chart suggested a greater impact of that feature on the model's prediction, increasing the predicted risk of carotid plaque. Conversely, a lower SHAP value implied a smaller impact on the prediction, decreasing the predicted risk. Figure 7 clearly shows how the top fifteen features with the highest contribution rates contribute to the model's predictions, simplifying the interpretation of complex model outputs and enhancing the understanding of the relationship between features and outcomes. Notably, age emerged as the paramount predictor in this study, followed by SBP, gender, LDL-C and CHOL.

## 4 Discussion

In the public health domain, seaching for efficient screening models for CAP is crucial. Han Z.et al discussed the significance of using integrative bioinformatics approaches and machine learning strategies to identify key genes and immune cell infiltration in the progression of carotid atherosclerotic plaques. By analyzing gene expression datasets and immune cell profiles, researchers can discover genes and immune cells that play critical roles in plaque development and progression (21). Cheng B.et al constructed eight models for predicting carotid plaques, with experimental results showing that the XGBoost algorithm outperformed other machine learning algorithms, achieving an AUC value of 0.808 (22). Cilla S et al. developed a CT angiography-based radiomics and machine learning model that effectively distinguished the vulnerability of carotid plaques (5). In contrast to previous studies that focused solely on genes, imaging, or clinical data, our research integrates various routine clinical indicators, physical examination data, and questionnaire results. This comprehensive approach allows for a more thorough assessment of factors influencing CAP, thereby enhancing the robustness and applicability of the model. Furthermore, building on prior research, we increased the sample size to over 19,000 participants. This large and diverse dataset overcomes the limitations of smaller sample sizes in previous studies, making the findings more reliable and generalizable. Among the seven machine learning algorithms we constructed, the LightGBM algorithm demonstrated the most outstanding performance, achieving an AUC of 0.85 in the validation cohort. This represents a significant improvement compared to other benchmark algorithms, such as XGBoost reported in the literature, highlighting the robustness and applicability of our machine learning model based on 21 features. The model utilizes routine physical examination data, making it highly practical in clinical settings. This helps in the early identification of high-risk populations and the timely implementation of preventive interventions. The implementation of such models can guide resource allocation in health policies and the development of targeted screening programs. For model performance evaluation, we employed ROC curves, calibration curves, and DCA curves, ensuring high reliability and accuracy of predictions. This multi-faceted evaluation method enhances the credibility of our findings and supports the broad applicability of our model across different population subsets. The results clearly indicate that the overall performance of the LightGBM model is high. This increases the credibility of the model's predictions and expands the generalizability of the research findings across the entire population. Subsequently, by employing the SHAP algorithm, our study provides a transparent and interpretable analysis of feature importance. A subsequent analysis of feature importance within the LightGBM framework revealed age as the quintessential determinant and gender, SBP total CHOL, and LDL-C as significant contributors to the model's predictive acumen.

In the contemporary landscape of predictive analytics, ML models stand at the forefront, eclipsing traditional statistical methods for their capacity to forge accurate predictive models from datasets characterized by limited size yet high-dimensional feature spaces. Despite their advanced capabilities, these models frequently face criticism for their lack of transparency, commonly referred to as the ‘black box’ issue, which obscures the understanding of their internal mechanisms (23). The deployment of the SHAP algorithm in this study effectively pierced this veil, enhancing model transparency by quantifying the influence of individual features on the predictive outcome. The importance of age, gender, SBP total CHOL, and LDL-C as pivotal determinants aligns with extant scholarly discourse (25–33). The prognostic significance of these factors is particularly salient in the context of carotid plaque, hypertension, and cardiovascular pathologies, where early detection is instrumental in risk mitigation. Age emerged as the preeminent feature, echoing findings that link advancing age with diminished vascular elasticity and a concomitant escalation in atherosclerotic propensity (25). As early as 2016, a cross-sectional study first reported the prevalence of CAP and its associated risk factors among adults aged 45 and above in rural China, highlighting significant gender differences. The study findings indicated that age, hypertension, diabetes, LDL-C, and CHOL were notably associated with the incidence of CAP. Importantly, it stressed that the prevalence among males was significantly higher than in females. This aligns with the findings of our research (26). Corroborating literature from The Lancet Global Health by Song et al. delineates an age-progressive increment in CAP incidence. Additionally, the study accentuates the influence of gender on the prevalence of carotid artery plaques, unequivocally indicating a higher susceptibility among males (2). In one study involving patients with type 2 diabetes, the prevalence of carotid atherosclerosis was significantly higher in the older age group, and the odds ratio for coronary heart disease (CHD) and stroke also increased with age. Gender differences are also evident; Males generally exhibit a higher prevalence of carotid atherosclerosis compared with females. In this study, the prevalence of carotid atherosclerosis was 58.18% in men and 51.54% in women, and the risk of coronary heart disease and stroke was correspondingly higher in men (27). Hypertension, specifically SBP—the arterial pressure during cardiac systole, is a significant factor contributing to arteriosclerosis. Persistently elevated SBP increases the pressure and tension on the vascular wall, making individuals prone to endothelial damage and the formation of arterial plaques. Long-term hypertension also leads to damage and dysfunction of the vascular wall, thereby promoting the development of arteriosclerosis (28). Arteriosclerosis, the primary pathophysiological process in the formation of carotid plaques, involves multiple mechanisms such as lipid deposition, inflammatory responses, cellular proliferation, and fibrosis. Hypertension accelerates this process by increasing the pressure and tension on the vascular wall, leading to plaque formation and growth. Additionally, hypertension can increase the risk of thrombosis by affecting blood flow and promoting platelet aggregation, further increasing the likelihood of severe complications (such as stroke) caused by carotid plaques. Li et al. conducted a population-based cross-sectional study that demonstrated hypertension as an independent risk factor for arteriosclerosis. Interestingly, when hypertension coexists with obesity, obesity may reduce the risk of arteriosclerosis caused by hypertension (28). The role of CHOL, particularly when present in excess, is well-documented in atherogenesis, with CHOL and LDL-C often vilified as “bad cholesterol and implicated in plaque accrual within critical vasculature” (24, 29–33). A previous study substantiates the robust association between elevated LDL-C levels and carotid plaque formation (24). Thus, this investigation confirmed the heightened importance of age, gender, SBP, CHOL, and LDL-C as features within the ML model, a conclusion supported by a wealth of evidence-based medical research. As early as 2011, Fang et al. evaluated age and plasma Oxidized Low-density Lipoprotein (ox-LDL) levels as potential risk factors and biomarkers for carotid plaque and stability based on carotid ultrasound results. The study concluded that plasma ox-LDL levels and age are potential risk factors for carotid plaque, with ox-LDL as a biomarker for screening vulnerable carotid plaque in clinical practice (24). However, Fang et al. lacked a comprehensive risk assessment compared to our study, which utilized comprehensive physical examination data. They did not account for all potential confounding variables influencing the relationship between ox-LDL and carotid plaque.

ML models for predicting CAP could assist in both clinical practice and health policy. Clinically, these models facilitate the early identification of individuals at high risk, enabling timely interventions that can lead to improved outcomes. In the realm of health policy, these models can guide the allocation of resources and the development of targeted screening programs, thereby enhancing healthcare efficiency and reducing the CVD burden. Furthermore, they support the creation of evidence-based cardiovascular guidelines and public health strategies aimed at mitigating risk factors. These models represent a shift towards a more proactive and personalized healthcare paradigm. The LightGBM machine learning model developed in this study demonstrated strong predictive performance, which carries significant public health implications. By leveraging routine physical examination data, doctors can effectively screen high-risk individuals for carotid plaque and implement preventive measures to avert the progression into more dangerous cardiovascular diseases. This approach not only enhances the efficiency of early intervention but also underscores the potential of utilizing big data technologies in public health.

Despite the encouraging findings, this study has certain limitations. First, the study's over 19,000 samples were all collected from the MJ Health Checkup Center in Beijing, indicating that our conclusions reflect only the characteristics of the local population. Therefore, it is currently unclear whether the established machine learning models can be generalized to the entire nation. To address this, it is recommended that future models be trained using a more extensive and diverse dataset. By incorporating data from various geographical locations and ethnic backgrounds, the generalizability and robustness of these models can be significantly improved. Secondly, the sensitivity of all models is not particularly high, which could result in some missed cases when used as screening tools. For example, many individuals with the condition might not be correctly diagnosed. We hope that future research can improve the overall performance of predictive models by using more training data and optimizing machine learning algorithms. This might involve exploring more advanced machine learning techniques, adjusting model parameters, or integrating novel data preprocessing methods to enhance sensitivity and reduce the omission rate. Furthermore, the generalizability of the models across different ethnic groups remains uncertain, as their validation relied on a 30% test set drawn from the pooled data rather than an independent external dataset. Future research and practical applications of CAP prediction models may encounter challenges that require enhancements. One major challenge is increasing the model's generalizability. To tackle this, future studies should include a broader and more diverse population sample from multiple regions and ethnic groups. This will help evaluate the model's performance across various demographics and potentially improve its predictive accuracy on a national scale. In summary, future research should focus on enhancing the model's generalizability and sensitivity. Simultaneously, practical applications should ensure seamless integration with healthcare systems, provide clear implementation guidelines, and conduct impact assessments to guarantee the model's effectiveness and ethical usage.

## 5 Conclusions

Our study demonstrated that ensemble learning models, particularly LightGBM, effectively predict the occurrence of CAP by analyzing factors such as gender, age, physical assessments, and blood biochemical markers. These models showcased high precision and consistent reliability, with age, gender, and SBP emerging as the most influential predictors. Utilizing these models, medical professionals can evaluate the risk of CAP using standard health checkup data rather than requiring ultrasound examinations. This advance significantly enhances the primary prevention of CVD. Future research should aim to improve the models’ performance and generalizability by incorporating data from a more diverse population.

## Data Availability

The raw data supporting the conclusions of this article will be made available by the authors, without undue reservation.
